# A Randomized Clinical Trial Comparing the Effects of Mindfulness-Based and Cognitive Behavioral Therapy-Based Stress Reduction in Medical Students

**DOI:** 10.1192/j.eurpsy.2024.698

**Published:** 2024-08-27

**Authors:** A. Yay Pençe, M. Çöldür, Z. Atalay, S. Aslan, B. Beba, C. Coşkun Sayın, I. Ekmekçi Ertek

**Affiliations:** ^1^Gazi University, Ankara; ^2^MEF University, İstanbul, Türkiye; ^3^Mindfulness Macedonia, slopje, North Macedonia; ^4^Mindfulness and Compassion Academy, İstanbul, Türkiye

## Abstract

**Introduction:**

Medical students face an enormous amount of stress (Dyrbye LN *et al*. *Ann Intern Med* 2008; **149:** 334-41). They suffer from higher rates of depression, anxiety, and suicide compared to the general population. Despite experiencing more mental health problems, there is a lack of research exploring ways to improve their mental health. Although there are a few small sample studies investigating the effectiveness of Mindfulness-Based Stress Reduction (MBSR) on medical students, there is no study comparing its effectiveness against an active intervention group in the literature (van Dijk I *et al*. *Acad Med* 2017; **92:** 1012-1021)

**Objectives:**

We aimed to compare the effects of the Mindfulness-Based Stress Reduction (MBSR) and the Cognitive Behavioural Based Stress Reduction (CBSR) group interventions on depressive and anxious symptoms and perceived stress of medical students.

**Methods:**

323 medical students applied to participate in one of the group interventions and were assessed with the Mini International Neuropsychiatric Interview. Of these, 253 (77% female, mean age=21.9 ± 2.9 years) were allocated into online MBSR (n=127) and online CBSR (n=126) groups after randomization. Their anxiety and depressive symptoms and perceived stress levels were assessed at baseline and after 8 weeks of interventions. 33,2% of participants (MBSR: n=39; CBSR: n=45) completed the protocol by attending five or more sessions. Both intention-to-treat (ITT) analysis and per-protocol (PP) analysis were used to assess outcomes. In the ITT analysis, we used multiple imputation to address missing values. All assessments and group interventions were done online.

**Results:**

In the ITT analysis, both MBSR and CBSR were found to be slight to moderately effective in reducing symptoms of depression (MBSR: d=.50; CBSR: d=.40), anxiety (MBSR:d=.73; CBSR: d=.52), and perceived stress (MBSR: d=.48; CBSR: d=.42), but they were no superior to each other. In the PP analysis, both interventions moderately to strongly improved the symptoms of depression (MBSR: d=1.03; CBSR: d=.74), anxiety (MBSR: r=-.74; CBSR: r=-.72), and perceived stress (MBSR: r=-.80; CBSR: r=-.68). While there was no statistically significant difference between them in reducing depressive symptoms and perceived stress, MBSR was found to be significantly more effective than CBSR in reducing anxiety symptoms (u=469, z=-2.756, p=0.006).

**Conclusions:**

Both MBSR and CBSR improve symptoms of depression and anxiety in medical students after 8 weeks of interventions. Completing the protocol or attending more sessions may increase the effectiveness of the interventions. While the interventions did not show superiority to each other in terms of effectiveness in reducing depressive symptoms and perceived stress, MBSR appears to be more effective in reducing anxiety symptoms compared to CBSR in the group that completed the protocol.

**Disclosure of Interest:**

None Declared

